# Color improves “visual” acuity via sound

**DOI:** 10.3389/fnins.2014.00358

**Published:** 2014-11-11

**Authors:** Shelly Levy-Tzedek, Dar Riemer, Amir Amedi

**Affiliations:** ^1^Department of Medical Neurobiology, The Institute for Medical Research Israel-Canada, Faculty of Medicine, The Hebrew University of JerusalemJerusalem, Israel; ^2^The Edmond and Lily Safra Center for Brain Sciences (ELSC), The Hebrew University of JerusalemJerusalem, Israel; ^3^The Cognitive Science Program, The Hebrew University of JerusalemJerusalem, Israel

**Keywords:** sensory substitution, visual rehabilitation, color perception, visual acuity, blind, visually impaired, visual cognition, human color vision

## Abstract

Visual-to-auditory sensory substitution devices (SSDs) convey visual information via sound, with the primary goal of making visual information accessible to blind and visually impaired individuals. We developed the EyeMusic SSD, which transforms shape, location, and color information into musical notes. We tested the “visual” acuity of 23 individuals (13 blind and 10 blindfolded sighted) on the Snellen tumbling-E test, with the EyeMusic. Participants were asked to determine the orientation of the letter “E.” The test was repeated twice: in one test, the letter “E” was drawn with a single color (white), and in the other test, with two colors (red and white). In the latter case, the vertical line in the letter, when upright, was drawn in red, with the three horizontal lines drawn in white. We found no significant differences in performance between the blind and the sighted groups. We found a significant effect of the added color on the “visual” acuity. The highest acuity participants reached in the monochromatic test was 20/800, whereas with the added color, acuity doubled to 20/400. We conclude that color improves “visual” acuity via sound.

## Introduction

Vision impairment imposes great challenges for approximately 285 million individuals worldwide (WHO, 2014). People who are blind or have low vision, face difficulties in recognition of objects, significantly impairing their ability to create a full representation of their environment.

Among the potential solutions aimed at overcoming these difficulties are sensory substitution devices (SSDs). These devices convey visual information via auditory or tactile input, thus making it possible for people who are blind or visually impaired to acquire information about the world that is not usually accessible through audition or touch. Visual-to-auditory SSDs are a relatively accessible solution, for they usually consist of a simple video camera that provides the visual input, a small computer running the conversion program and stereo headphones that provide the resulting sound patterns to the user (e.g., Meijer, 1992; Levy-Tzedek et al., 2012a; Abboud et al., 2014).

The human visual pathway engages in recognizing external objects and judging their form, color, and movement; in what is known as “the binding problem,” different features of an object are analyzed by disparate brain areas, and then combined to create a single Gestalt-like percept (Cronly-Dillon et al., 1999). This neural pathway is accessible to other, non-visual, modalities for performing some of these functions (e.g., Striem-Amit et al., 2012b). By using parts of this neural substrate, SSDs can be used to recognize the shape, identity, and location of objects. This ability has been demonstrated in several studies; for example (Amedi et al., 2007) reported activation in a tactile-visual shape area when shape information was conveyed via a visual-to-auditory SSD. Renier et al. (2005) reported activation in occipito-parietal and occipito-temporal areas when participants were asked to perform a depth-perception task with a visual-to-auditory SSD. Striem-Amit et al. (2012b) demonstrated a double dissociation between the ventral and dorsal functional areas when listening to auditory representations of images.

We developed a visual-to-auditory SSD, named the EyeMusic (Levy-Tzedek et al., 2012a,b; Abboud et al., 2014), which transforms digital images into soundscapes, or auditory representations of images, in a manner similar to the vOICe SSD developed by Meijer (1992). These soundscapes, however, are composed of musical notes, rather than pure tones, each corresponding to a pixel on the original image. In another study we conducted, participants rated the extent to which it was pleasant to listen to the EyeMusic's soundscapes as 3.6 on average, on a scale of 1–5; Twenty one out of 23 participants in that study found the soundscapes of the EyeMusic to be more pleasant to listen to than to the vOICe system (Abboud et al., 2014). The image is scanned from left to right, and columns of pixels are played out sequentially. The time elapsed since the beginning of the scan indicates the x-axis location of the pixel: pixels that are further on the left are sounded out earlier than pixels which are on the right of the image. The height of the pixel along the y-axis determines the pitch of the musical note representing it: the higher is the pixel, the higher is the pitch of the note representing it. The brightness of the pixel determines the sound volume of the musical note: the brighter the pixel, the higher the volume. Another novelty of the EyeMusic is the use of different musical instruments to represent each of five colors (red, green, blue, yellow, and white), with the black color represented by silence. The EyeMusic's algorithm employs a clustering routine, which creates an image with these six colors (Abboud et al., 2014).

In an effort not to overwhelm the auditory system with multiple simultaneous notes played all at once, the EyeMusic was designed to represent images at an intermediate resolution of 40 × 24 pixels. At the same time, color information was included as a means to augment the *effective resolution*, thereby increasing acuity levels, based on the findings that color can improve object recognition (e.g., Wurm et al., 1993; Boucart et al., 2008; Lloyd-Jones and Nakabayashi, 2009). In this study, we set out to test the hypothesis that color information, conveyed via sound, can increase “visual” acuity of images conveyed via the EyeMusic SSD.

**Figure 1:**
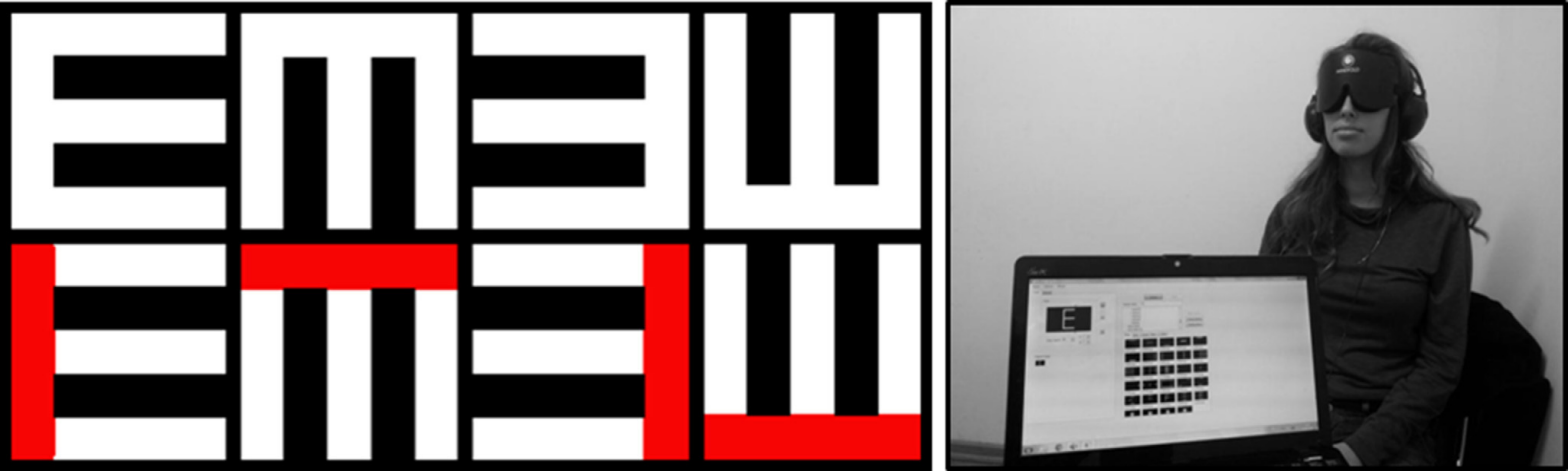
**Left:** The Snellen tumbling-E stimuli that were auditorily presented to the participants via the EyeMusic SSD. Top: all-white stimuli; Bottom: red-and-white stimuli. **Right:** The experimental setup. A blindfolded sighted participant is hearing, via a pair of headphones, the soundscape representing the upright letter “E,” generated by the EyeMusic software installed on a laptop computer.

In recent years, several studies have quantified the “visual” acuity of blind and/or blindfolded sighted individuals perceiving images via SSDs by using a standard ophthalmological test: the Snellen tumbling-E test (Sampaio et al., 2001; Chebat et al., 2007; Striem-Amit et al., 2012c), where the participants are asked to indicate which direction the capital letter “E” is facing: up/down/left/right (see Figure 1). We used this test to quantify the “visual” acuity of 13 blind and 10 blindfolded sighted individuals using the EyeMusic SSD. We employed two forms of the Snellen test: in one, the letters were monochromatic (white), and in the other, the letter “E” was composed of two colors (red and white, see Figure 1 Left).

## Methods

### Participants

A total of 23 individuals participated in this experiment. Thirteen blind individuals (6 male, 7 female; age 32 ± 9 years (mean ± *SD*), of which 10 are congenitally blind, 2 early blind and 1 partially blind) and 10 blindfolded sighted participants (3 male, 7 female, 26 ± years). All sighted participants were naïve to the use of SSDs, and three of the blind participants have not previously been trained on the EyeMusic SSD. Ten of the 13 blind participants had been training with the EyeMusic for a variable amount of time, roughly between 4 and 80 h. The Hebrew University's ethics committee for research involving human subjects approved the experimental procedure and written informed consent was obtained from each participant. An additional blind participant was trained but not tested, as he was not able to perform the task. All participants were seated such that the computer screen was facing away from them, as demonstrated in Figure 1.

### Stimuli and procedure

The experimental set-up consisted of a laptop running the EyeMusic and a pair of headphones through which the soundscapes were conveyed to the participants. The sighted participants wore a blindfold (see Figure 1). Each participant took part in one session, which lasted approximately 1.5 h on average, and consisted of a training session and an experimental session. The training session lasted 22 ± 7 min (mean ± *SD*), and consisted of five phases:

*Training phase I*: The participants were taught the basics of interpreting visual information that is conveyed by sound using the EyeMusic algorithm. An auditory cue (beep) was sounded at the beginning of each left-to-right scan of the image. It was explained to the participants that (1) the higher musical notes represent pixels that are located higher on the y-axis of an image, (2) the timing of the sound after the cue indicates the x-axis location of the pixel (that is, an object that is located on the left of the image will be heard earlier than an object located on the right), and (3) different colors are represented by different musical instruments. The participants learned to decipher soundscapes representing simple white lines of various lengths and thicknesses in two orientations: horizontal and vertical. Horizontal lines are characterized by a repetition of the same note, since the location of the pixel on the y-axis determines the pitch of the note representing it; vertical lines are a set of differently pitched musical notes which are played simultaneously for a short period of time.
*Training phase II*: Once the participants mastered the recognition of these simple lines, combinations of lines were presented, eventually constructing the letter “E.”
*Training phase III*: The Snellen tumbling-E in four directions (up/down/left/right; see Figure 1 Left, top row) was introduced, portrayed in a size larger than the sizes that were later tested in the experimental session.
*Training phase IV*: The red color was introduced, by playing the soundscapes of a few simple lines (horizontal and vertical) in red so that the participants would become acquainted with its different musical timber (the white color is played by a choir while the red color is played by a synthesized organ).
*Training phase V*: Participants were trained on discerning the orientation of the large, monochromatic, white-only Snellen “E”s (introduced in training phase III) until they reached learning criterion of six successive correct responses.

For the blind participants, the order of training phases IV and V was reversed.

Upon completion of the training session, the experimental session was conducted.

#### Stimuli

We used stimuli identical to those used in Striem-Amit et al. (2012c) for the white-only “E”s (top row in Figure 1). The stimuli were created by photographing a standard Snellen chart with a 66° field-of-view webcam (A4Tech, Montclair, CA, USA) from a distance of 1 m, and calculating the Snellen fraction from this distance according to the standard reference scale. In addition, we created a second set of stimuli in which the letter E was composed of two colors—red and white—such that the “backbone” of the letter E is colored red (see Figure 1, bottom row). With these two-color stimuli, the participants have additional information regarding the location of the E's backbone, as it is portrayed using a different musical instrument, compared to the rest of the E's components. Hearing the red color (or timbre) first, and only then the white color (timbre), would help the participant identify that the E is upright, facing the right (the first E from the left in Figure 1); Hearing both colors (timbres) at the same time, but the red represented by higher-pitched notes than the white, would indicate the E is rotated and facing downwards (the second E from the left in Figure 1), and so forth.

The decrease in the size of the letters used in each acuity level was translated into an audio signal that was shorter in duration (along the x-axis), and spanned a smaller range of musical notes (in the y-axis), such that the notes were much closer to each other in pitch. These changes in the auditory domain, created by the EyeMusic algorithm, reflected the changes in the visual stimuli in the various orientations. See Supplementary Materials for sample soundscapes.

#### Experimental session

The experimental task consisted of two sessions of the Snellen tumbling-E test: one of white-only E's and another one of red-and-white E's. The order of the two sessions was counterbalanced across participants. The participants were told that their task is to name the orientation of the “E” (i.e., up, down, left, or right) and that the stimuli will be presented in a decreasing order of size (see Table 1). They were told that since the “E” is growing smaller it is possible that at a certain point they will not be able to make a reasoned choice regarding its orientation, and that in such a case they should guess. There were nine letter sizes, and each of the orientations was presented four times in each size (for a total of 144 trials: 9 sizes × 4 orientations × 4 repetitions). No “zoom in” of the soundscapes via the software was permitted, thus the field-of-view was fixed during the entire experiment to represent a 66° visual field (much more than the WHO blindness threshold for field of view, which is 10°). The soundscape of the stimulus was played until the participant responded regarding its orientation (no feedback was given to the participants during the testing). Both the answer and the reaction time were recorded and analyzed.

**Table 1:** **Snellen stimuli sizes, reported as Snellen fractions (distance from which the participant perceives the letter in meters in the numerator and the distance from which a normally sighted individual would perceive the same letter in the denominator), the physical letter size in mm, and logMar, a linear scale which expresses the logarithm of the minimal angle of resolution**.

**Snellen acuity**	**Actual letter size (mm)**	**logMAR**
20/1600	117	1.903
20/1400	102	1.845
20/1200	88.7	1.778
20/1000	73	1.699
20/800	58	1.602
20/600	44	1.477
20/500	36	1.398
20/400	29	1.301
20/360	26	1.255

### Data exclusion

One blind participant, after meeting the training criterion, said at the start of the experimental session (when tested on the largest letter size) that he needed further instruction. Therefore, the data from the largest letter size for this participant (both color and white stimuli) were excluded from the analysis. During the experimental session of another participant, a technical error caused the skipping of a single repetition of the second-largest letter, with the colored stimulus. Therefore, 15 trials were analyzed for this letter size in color for this participant, rather than 16.

### Statistical analysis

Since data collected were binary, we used logistic regression analysis within the framework of a general linear mixed model (GLMM), where the group, letter size, and color were the fixed effects, and the individual participants were treated as a random effect. The final model was achieved by using a backward-elimination algorithm. Significance level was set at 5% for all statistical analysis. SPSS 20 software was used for the analysis.

## Results

Average reaction time per stimulus across the different sizes was 8.4 ± 2.0 s in the white-only Snellen test and 8.6 ± 4.2 s in the red-and-white Snellen test.

No significant three-way interaction was present between group, color, and letter size [*F*_(8, 376)_ = 0.956, *p* = 0.47]. There was no interaction between group and color [*F*_(1, 384)_ = 0.037, *p* = 0.848].

No statistically significant order effects were present [*F*_(1, 384)_ = 3.0, *p* = 0.083]. That is, it did not matter whether the white-only task was performed first or the white-and-red task was performed first.

All main effects (group, color, and letter size) were found to be significant, as well as the interaction between letter size and group, and the interaction between letter size and color (see the result of the final logistic regression model following backward elimination in Table 2).

**Table 2:** **Statistical analysis**.

	***F***	***df*1**	***df*2**	***p*-value**
Group (sighted/blind)	4.5	1	385	0.035
Size of stimulus	169.4	8	385	<0.001
Color (white-only/red and white)	79.1	1	385	<0.001
Group × size interaction	2.6	8	385	0.009
Size × color interaction	14.5	8	385	<0.001

*Results for the fixed effects from the final logistic regression model following backward elimination*.

**Figure 2:**
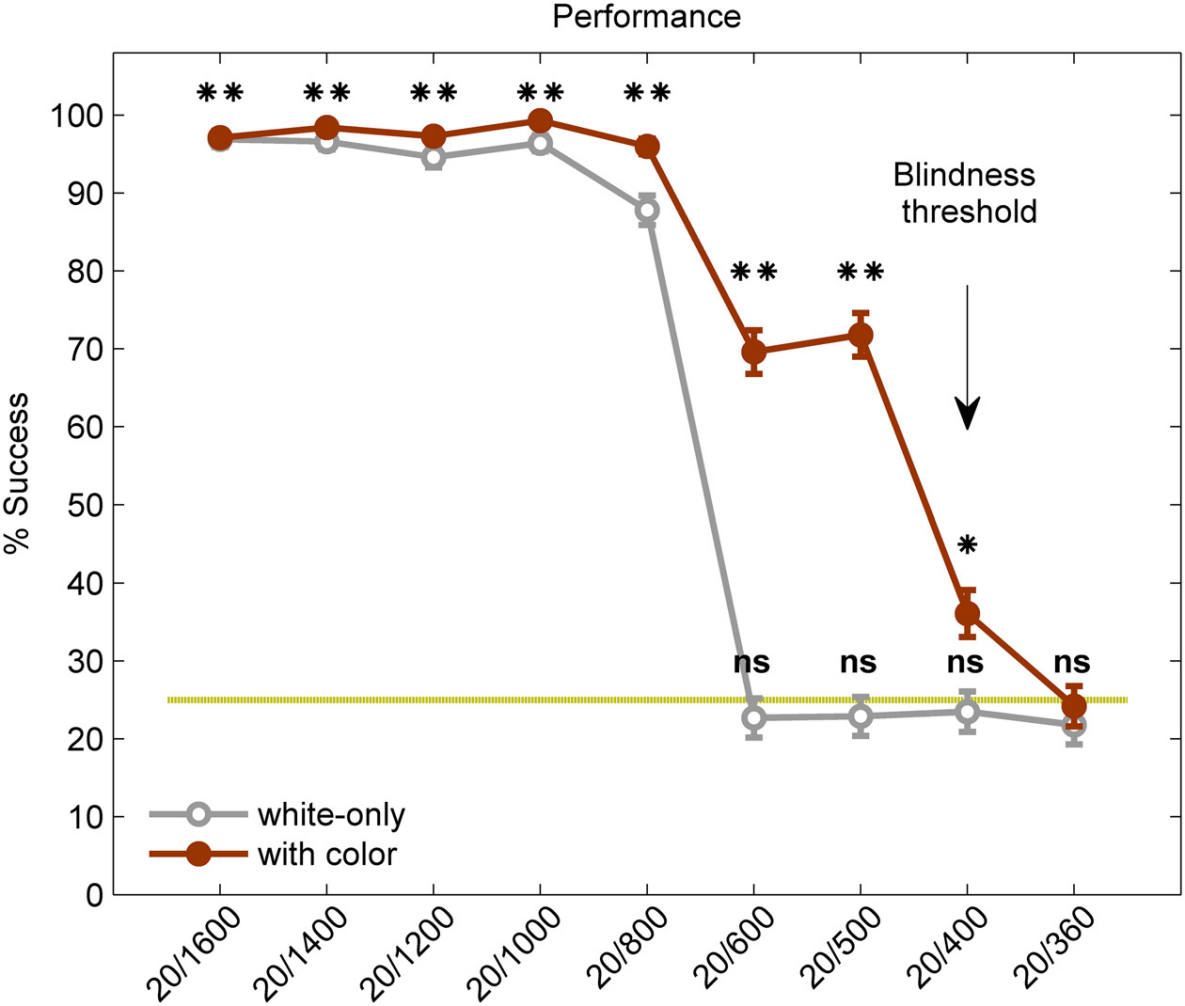
**“Visual” acuity of the blind and sighted participants using a visual-to-auditory SSD—with and without color**. Overall performance on the Snellen E test: results from the white-only task (gray line) and from the white-and-red task (red line). Asterisks represent difference from chance level (^**^p < 1 × 10e^−10^, ^*^p = 0.0001, ns, not significant). Chance level (25%) is marked by broken green line. The World Health Organization (WHO) criterion for blindness, at an acuity level of 20/400 (the blindness threshold), is marked for reference with an arrow. The error bars represent standard error.

For the white-only stimuli, the highest acuity for which success rate was significantly higher than chance (25%) was 20/800 [one-tailed *t*-test (*df* = 385), *p* < 1e^−10^, Figure 2, gray line, and Table 3]. For the colored stimuli, however, the highest acuity for which success rate was significantly higher than chance was 20/400 [one-tailed *t*-test (*df* = 385), *p* = 0.0001, see Figure 2, red line, and Table 3]. In other words, acuity with color was twice the acuity without added color information.

**Table 3:** **Performance compared to chance level (25%)**.

**Snellen size**	**Percent success—with color (%)**	**Difference from chance (*p*-value)—with color**	**Percent success—white-only (%)**	**Difference from chance (*p*-value)—white-only**
20/1600	97.1	*p* < 1 × 10e^−10^	96.9	*p* < 1 × 10e^−10^
20/1400	98.4	*p* < 1 × 10e^−10^	96.6	*p* < 1 × 10e^−10^
20/1200	97.3	*p* < 1 × 10e^−10^	94.6	*p* < 1 × 10e^−10^
20/1000	99.3	*p* < 1 × 10e^−10^	96.4	*p* < 1 × 10e^−10^
20/800	96.0	*p* < 1 × 10e^−10^	87.8	*p* < 1 × 10e^−10^
20/600	69.6	*p* < 1 × 10e^−10^	22.7	0.82
20/500	71.8	*p* < 1 × 10e^−10^	22.9	0.80
20/400	36.1	0.0001	23.5	0.72
20/360	24.2	0.62	21.8	0.90

Examining the effects of color within each letter size reveals a significant effect of color for stimuli in sizes 20/400-20/1000 (see Table 4 for details).

**Table 4:** **Improvement in performance due to the addition of meaningful color information**.

**Stimulus size**	**Improvement in performance due to color (%)**	***p*-value [two-tailed *t*-test (***df*** = **385**)]**
20/400	12.6	*p* < 0.001
20/500	48.9	*p* < 0.001
20/600	46.9	*p* < 0.001
20/800	8.3	*p* < 0.001
20/1000	2.9	*p* = 0.007

**Figure 3:**
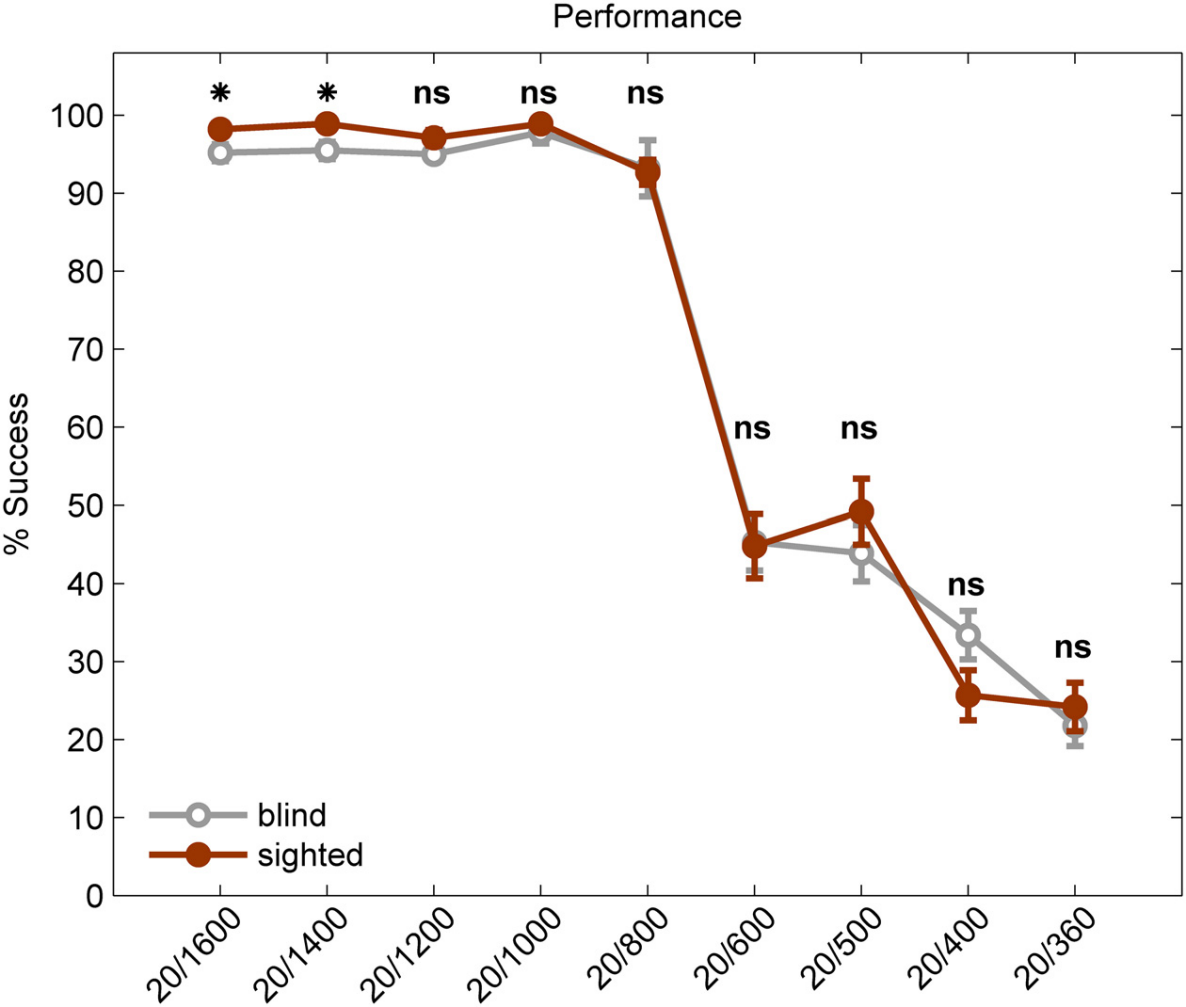
**Visual Acuity of the blind vs. the sighted participants using a visual-to-auditory SSD**. Performance of blind participants (gray line) and sighted participants (red line) on the Snellen E test. An asterisk marks a significant difference between the two groups (*p* < 0.05; ns, not significant). The error bars represent standard error.

*Post-hoc* analysis on the interaction between group and letter size revealed that the difference between blind and sighted was only in the two largest letter sizes. The sighted performed better than the blind by 3.4% on average for stimulus size 20/1400 [two-tailed *t*-test (*df* = 385), *p* = 0.006], and by 2.9% in stimulus size 20/1600 [two-tailed *t*-test (*df* = 385), *p* = 0.036; see Figure 3].

Anecdotally, several of the blind participants reported that they imagined the letter and its components in their mind's eye during the experiment, and one congenitally blind participant noted that the color served as a highlighter in her experience.

## Discussion

In this study, we demonstrated that users of the EyeMusic SSD, both blind and sighted, were able to achieve a “visual” acuity of 20/800 when presented with monochromatic letters, and the acuity doubled to 20/400 when the presented letters were composed of two colors. The EyeMusic conveys visual information via sounds, and the two colors are depicted by two different sound elements: the white color is represented by a choir, and the red color by notes recorded from a synthesized organ. This report is the first, to the best of our knowledge, to report the effect of color information on “visual” acuity via an SSD.

We have used the color feature of the EyeMusic in previous works. However, in Abboud et al. (2014) it was used to study how well users could distinguish among the different colors represented by different musical timbers, and in Levy-Tzedek et al. (2012a,b) it was used to distinguish between two functional items (one color marked the desired destination of a movement, and the other color marked the actual position at the end of the movement). To the best of our knowledge, the current experiment is the first to add color information as a meaningful addition to the identification of an object's orientation in space, and the first to study the effect of color on “visual” acuity when images are represented via sounds.

The acuity results reported here supersede those achieved via a tactile SSD, the tongue display unit (TDU) (20/430 and 20/1800 in Sampaio et al., 2001 and Chebat et al., 2007, respectively), and those attained by currently available retinal prostheses [20/1000, for a 38 × 40 pixels array (Zrenner et al., 2011); see Table 5]. These results are comparable (though slightly lower) than those achieved with the vOICe SSD; we have previously shown, using the vOICe SSD, with a resolution of 25,344 pixels, that participants reached an acuity of 20/320 (Striem-Amit et al., 2012c). Importantly, the resolution of the EyeMusic is 26 times lower than that of the vOICe. And so, with a much lower pixel resolution, but with the addition of color information, it was possible to achieve comparable acuity levels with the two devices.

**Table 5:** **Acuity levels reached on the Snellen test with three SSDs**.

**Device**	**Study**	**Device resolution (pixels)**	**Color information?**	**Sensory input**	Acuity^*^
TDU	Sampaio et al., 2001	144	No	Tactile	20/860-20/430
TDU (BrainPort)	Chebat et al., 2007	100	No	Tactile	20/1800
Retinal prosthesis	Zrenner et al., 2011	1520	No	Retinal stimulation	20/1000
The vOICe	Striem-Amit et al., 2012c	25,344	No	Auditory	20/320
The vOICe	Haigh et al., 2013	11,264	No	Auditory	20/13965-20/2464
The EyeMusic	Current report	960	Yes	Auditory	20/400

* *Acuity: the highest acuity reached by users of the device, as reported in these studies; Note that the best acuity level achieved was determined using different methods in these various studies*.

Importantly, while in the current study, the resolution of the EyeMusic was 40 × 24 pixels, a newer version of the EyeMusic has been developed, which features an increased resolution of 50 × 30 pixels (Maidenbaum et al., 2014). We anticipate that the new version, which combines a higher resolution with color information, will enable users to achieve even higher functional acuity levels. We plan to test this in future work.

It should be noted that despite reports of compensatory advantage in auditory processing in blind individuals (Gougoux et al., 2004; Collignon et al., 2009; Hotting and Roder, 2009), and despite some of the blind participants having prior experience with the EyeMusic software, we found a slight advantage of the sighted group compared to the blind group (by ~3% in the two largest letter sizes, see Figure 3), possibly due to the familiarity of the sighted group with the letter E.

A recent literature review and meta-analysis which included 35 independent experiments, comprising 1535 participants concluded that color information contributes to object recognition, not only in photographs, but also in line drawings (Bramao et al., 2011). Indeed, color has been demonstrated to play an important role in object and scene recognition in various conditions, e.g., when images have a low resolution (Wurm et al., 1993; Torralba, 2009), in speeded scene recognition (Oliva and Schyns, 2000; Rousselet et al., 2005; Castelhano and Henderson, 2008), object recognition (Lloyd-Jones and Nakabayashi, 2009), and in individuals with age-related macular degeneration (Boucart et al., 2008). Our results demonstrate that color represented by timbre also improves functional “visual” acuity when image information is conveyed via sound. Here, we demonstrate that color (represented by timbre) can be used in a device with intermediate resolution (960 pixels for the EyeMusic) to increase the *effective resolution* afforded by it.

These results demonstrate that high visual acuity can be achieved by early-onset and congenitally blind individuals even after decades of (or life-long) blindness, via sensory substitution. They are in line with evidence of adult plasticity at the level of functional “visual” perception in the adult congenitally blind (Hamilton and Pascual-Leone, 1998; Reich et al., 2011).

We have previously demonstrated (Striem-Amit et al., 2012a,b; Striem-Amit and Amedi, 2014) with the vOICe SSD that, following extensive training, visual-to-auditory SSDs specifically activated task-related areas in the occipital lobe, in addition to non-specific activation in the auditory cortex, even in congenitally blind individuals, with no prior visual experience. These results suggest that the responses of the participants are not merely based on retrieval of learned paired-associate learning between the sounds and the shapes they represent. The specific location of activation within the visual stream depends on the specific task: we have shown that the same stimuli, delivered via the vOICe SSD, activated either the ventral or the dorsal stream within the occipital lobe, depending on whether the congenitally blind participants were asked to identify the object presented, or determine its location, respectively (Striem-Amit et al., 2012b). We are currently in the process of studying the brain activation patterns in response to color that is conveyed via timbre. While it is reasonable to assume that initially the perception of color via the EyeMusic is an explicit cognitive task, expressly associating between timbers and colors, our experience so far has provided evidence to support a holistic interpretation of the auditory input following extensive training. For example, if a simple pair association were formed for specific sounds, shapes, and colors, we would anticipate that in more complex natural settings, deciphering high-acuity information from sounds may be prohibitively difficult and slow. And yet, users of the EyeMusic have been able to use a live camera to capture the surrounding visual scene in real time, and respond to the “visual” input in an appropriate and accurate manner (e.g., reach for a red apple in a bowl of green apples; see a video demonstration in https://www.youtube.com/watch?v=wlWawfCUMsU, movie S1 in Abboud et al., 2014).

It has been previously suggested that SSDs may be used as a complement to visual prostheses (Striem-Amit et al., 2012c). For example, they can be used before a retinal prosthesis implantation, to train the visual cortex to “see” again after years or life-long blindness, by utilizing the preserved “visual” task functionality of the occipital cortex of the blind (e.g., Striem-Amit et al., 2012b). Similarly, they can be used post-operatively, to provide an explanatory signal—or a “sensory interpreter”—in parallel to the visual signal arriving from the prosthesis, as early-onset blind individuals may otherwise find it difficult to interpret direct visual signals (Fine et al., 2003; Gregory, 2003). It can also add details which are not otherwise provided by the prosthesis. The current experiment suggests that color information may well add to the benefit of pre- and post-surgical training with SSDs, as part of a comprehensive clinical visual rehabilitation protocol.

## Author contributions

Amir Amedi conceived the experiment; Amir Amedi and Shelly Levy-Tzedek supervised the project; Dar Riemer acquired the data; Shelly Levy-Tzedek analyzed the data; Shelly Levy-Tzedek and Dar Riemer wrote the paper; Amir Amedi critically reviewed the paper, and provided financial support.

### Conflict of interest statement

Author Amir Amedi is among the holders of the patent: Amedi, Amir, and Shlomo Hanassy. “Representing visual images by alternative senses.” U.S. Patent Application 13/505,642, filed November 3, 2010. The other authors declare that the research was conducted in the absence of any commercial or financial relationships that could be construed as a potential conflict of interest.
