# Supplementary material for: Color improves “visual” acuity via sound
Source: Front Neurosci. 2014 Nov 11;8:358. doi: 10.3389/fnins.2014.00358 (PMC4227506; doi:10.3389/fnins.2014.00358)
Supplement: Supplementary file 1 [file DataSheet1.DOCX]

These are sample soundscapes of some of the stimuli – both white-only and white-and-red – recorded by the EyeMusic software, v. 4.5.6, at a 40x24 resolution. The soundscapes are of stimuli used for Snellen acuity levels of 20/1600, 20/800 and 20/400, each in both color options and in the four possible orientations.

The following are the descriptions of the stimuli that the audio files correspond to:

Audio1 – Snellen size 20/400, Down, color (red + white)

Audio2 – Snellen size 20/400, Left, color (red + white)

Audio3 – Snellen size 20/400, Right, color (red + white)

Audio4 – Snellen size 20/400, Up, color (red + white)

Audio5 – Snellen size 20/800, Down, color (red + white)

Audio6 – Snellen size 20/800, Left, color (red + white)

Audio7 – Snellen size 20/800, Right, color (red + white)

Audio8 – Snellen size 20/800, Up, color (red + white)

Audio9 – Snellen size 20/1600, Down, color (red + white)

Audio10 – Snellen size 20/1600, Left, color (red + white)

Audio11 – Snellen size 20/1600, Right, color (red + white)

Audio12 – Snellen size 20/1600, Up, color (red + white)

Audio13 – Snellen size 20/1600, Down, white-only

Audio14 – Snellen size 20/1600, Left, white-only

Audio15 – Snellen size 20/1600, Right, white-only
